# Authors’ Response to Peer Reviews of “Assessing the Limitations of Large Language Models in Clinical Practice Guideline–Concordant Treatment Decision-Making on Real-World Data: Retrospective Study”

**DOI:** 10.2196/84173

**Published:** 2025-11-03

**Authors:** Tobias Roeschl, Marie Hoffmann, Djawid Hashemi, Felix Rarreck, Nils Hinrichs, Tobias Daniel Trippel, Matthias I Gröschel, Axel Unbehaun, Christoph Klein, Jörg Kempfert, Henryk Dreger, Benjamin O'Brien, Gerhard Hindricks, Felix Balzer, Volkmar Falk, Alexander Meyer

**Affiliations:** 1 Department of Cardiology, Angiology and Intensive Care Medicine, Deutsches Herzzentrum der Charité, Berlin, Germany; 2 Charité – Universitätsmedizin Berlin, corporate member of Freie Universität Berlin and Humboldt-Universität zu Berlin, Charitéplatz 1, Berlin, 10117, Germany, 49 17632864219; 3 Berlin Institute of Health at Charité – Universitätsmedizin Berlin, BIH Biomedical Innovation Academy, BIH Charité Digital Clinician Scientist Program, Berlin, Germany; 4 DZHK (German Centre for Cardiovascular Research), partner site Berlin, Berlin, Germany; 5 Department of Cardiothoracic and Vascular Surgery, Deutsches Herzzentrum der Charité (DHZC), Berlin, Germany; 6 Department of Infectious Diseases and Respiratory Medicine, Charité – Universitätsmedizin Berlin, Berlin, Germany; 7 Department of Cardiac Anesthesiology and Intensive Care Medicine, Deutsches Herzzentrum der Charité (DHZC), Berlin, Germany; 8 Department of Perioperative Medicine, St Bartholomew’s Hospital and Barts Heart Centre, London, United Kingdom; 9 Charité – Universitätsmedizin Berlin, Institute of Medical Informatics, Berlin, Germany; 10 Department of Health Sciences and Technology, Translational Cardiovascular Technologies, Institute of Translational Medicine, Swiss Federal Institute of Technology, Zürich, Switzerland; 11 Berlin Institute for the Foundations of Learning and Data – TU Berlin, Berlin, Germany

**Keywords:** large language model, foundation model, reasoning model, treatment decision-making, aortic stenosis, clinical practice guidelines, medical data processing

*This is the authors’ response to peer-review reports for “Assessing the Limitations of Large Language Models in Clinical Practice Guideline–Concordant Treatment Decision-Making on Real-World Data: Retrospective Study.”*

## Round 1 Review

### Reviewer K [1]

*1. To improve the discussion on bias in large language models (LLMs) for clinical decision-making, the study [2] should include the following aspects:*

*If LLMs are trained predominantly on Western medical literature or specific demographic groups, their recommendations may not generalize well to diverse patient populations. If the data used to fine-tune the model lack representation from certain ethnic, gender, or socioeconomic groups, the artificial intelligence may produce recommendations that are not universally applicable. Even with a diverse dataset, biases can arise due to model architecture, reinforcement learning strategies, or human-in-the-loop feedback mechanisms that shape model responses.*

**Response:** Thank you for this thoughtful and important comment. We fully agree that the generalizability and fairness of LLMs in health care are significantly influenced by the composition of their training and fine-tuning data. As you rightly note, underrepresentation of certain ethnic, gender, or socioeconomic groups can lead to biased outputs and potentially widen existing health disparities. Indeed, we have also discovered, for example, bias toward transcatheter aortic valve implantation in our experiments, as indicated through the Frequency Bias Index in Figure 2 and Table S9. All LLMs were taken off-the-shelf without fine-tuning as the cohort size was limited by the inherently low incidence of eligible cases and the stringent requirements for high-quality, comprehensive patient data. Each case required detailed manual review and the generation of structured case summaries, which further constrained the pool of analyzable data. As a result, stratification and investigation of bias by additional features such as ethnic, gender, or socioeconomic features was not feasible. In the Limitations section, we have added that potential biases remain unaddressed.

*2. What datasets were used? If real patient data were used, specify its source (eg, electronic health records, clinical trial data, or synthetic datasets). Provide the total number of cases or records used for testing the large language models. If synthetic data were generated, describe the method used to create the data. Were diverse age groups, genders, and ethnic backgrounds represented? A lack of diversity in data can affect the generalizability of results.*

**Response:** Thank you for addressing this very important point. As described in the Methods section, we have used real clinical reports in PDF format from our hospital information system and extracted the content into text files. Either these text files (experiments RAW and RAW+) or manually drafted summaries (SUM and SUM+) from these text files had been used as input to the LLMs. No trial or synthetic data were used.

*3. What datasets were used? If real patient data were used, specify its source (eg, electronic health records, clinical trial data, or synthetic datasets). Provide the total number of cases or records used for testing the large language models. If synthetic data were generated, describe the method used to create the data. Were diverse age groups, genders, and ethnic backgrounds represented? A lack of diversity in data can affect the generalizability of results.*

**Response:** Thank you for your comment. This comment is identical to Comment #2, which we have addressed in detail above. To summarize: we used real clinical reports extracted from our hospital information system (electronic health records), and no synthetic or trial data were used. Additional details, including data source and sample characteristics, are provided in our response to Comment #2 and in the revised Methods section under “Study Population” and “Data Collection and Preprocessing.”

*4. The study’s impact can be significantly enhanced by addressing the following challenges: Raw medical reports often include free-text narratives, physician notes, abbreviations, and inconsistencies, requiring advanced natural language processing techniques such as entity recognition, text normalization, and standardization. These reports may also contain irrelevant information, redundancies, or nonessential clinical details. Effective preprocessing is essential to filter out unnecessary content while preserving critical medical insights. A key consideration is how to optimize this preprocessing to mitigate these challenges efficiently.*

**Response:** Thank you for this insightful comment. The central objective of our study was to assess model performance using the same type of raw clinical data that health care professionals routinely encounter, including free-text narratives and unstructured content. The rationale behind this approach was that, for real-world clinical implementation, it would be most beneficial if LLMs could generate guideline-concordant treatment recommendations directly from routine clinical documentation—without relying on curated or heavily preprocessed inputs. This would help avoid the considerable time and resource demands associated with manual or automated preprocessing pipelines. To explore this, we compared model performance on raw clinical reports with performance on highly preprocessed, structured synopses, as used in previous studies where frontier models have shown strong results. We simulated this optimized input scenario through manually drafted summaries (SUM and SUM+), which represent a best-case input condition. Replicating such preprocessing through automated means would require extensive quality control mechanisms and may still fall short of the accuracy and relevance achieved through expert curation.

### Reviewer BI [3]

*1. The format and provenance of the SUM (“case summary”) reports require clearer specification. Although the authors note these summaries were “manually generated,” it would be helpful to state whether they followed a standardized template, who exactly drafted them (eg, experienced cardiologists, research assistants), and which elements of the Heart Team protocol they distilled into each summary.*

**Response:** Thank you for pointing this out. We agree that this aspect was not sufficiently described in the original manuscript. We have revised the Methods section under “Experiments” to clarify that the case summaries were manually created but adhered to a structured format: all patient characteristics documented in the heart team protocol were systematically addressed by either affirming, negating, or populating them with patient-specific values. An illustrative example is provided in Table S6.

*2. The authors report that the original medical documents were saved as PDFs and later converted to plain text. It would be helpful to clarify this process to avoid confusion, since LLMs accessed via chat interfaces or application programming interfaces often struggle with PDF inputs or text embedded in images, treating them differently from pure text. A brief discussion acknowledging this limitation—and explaining how PDF parsing was handled or validated—would help readers assess real-world applicability.*

**Response:** We appreciate the reviewer’s helpful comment. In every case, plain text—not PDF files—was provided as input. To clarify this point in the Methods section, we have added a description of the process: the text content of each PDF file was programmatically extracted using the Tesseract OCR software and concatenated into a single plain-text file, which was then used as input for the models for the RAW and RAW+ experiments.

*3. Raw inputs (PDFs and summaries) were provided in German (except for BioGPT, which required translation to English). A comment in the Discussion about how model performance can vary by input language—perhaps citing studies that showed different results in Polish versus English—would contextualize the findings for non-English clinical settings:*

- *Rosoł M, Gąsior JS, Łaba J, Korzeniewski K, Młyńczak M. Evaluation of the performance of GPT-3.5 and GPT-4 on the Polish Medical Final Examination.* Sci Rep. *2023;13(1):20512.*

**Response:** We appreciate the reviewer’s thoughtful suggestion. We agree that input texts in languages other than English may pose an additional challenge for LLMs, as they are primarily trained on English-language literature. We have added a comment and the suggested citation in the Limitations section. The study you cited suggests that more recent GPT models may be more language-agnostic than previous generations, though it remains unclear whether this holds true for other languages and frontier models.

*4. The Discussion section feels comparatively weak and could be strengthened by broader literature coverage. For instance, a brief discussion of input formats—pure text versus multimodal inputs—would be valuable, especially given the inclusion of GPT-4o, which handles images. Preliminary studies in this area include:*

- *Günay et al. Comparison of emergency medicine specialist, cardiologist, and ChatGPT in electrocardiography assessment. Am J Emerg Med. 2024 Jun;80:51-60.*
- *Zeljkovic et al. Beyond text: the impact of clinical context on GPT-4’s 12-lead electrocardiogram interpretation accuracy. Canadian J Cardiol. 2025 Jul;41(7):1406-1414.*

*These compare electrocardiogram interpretation with and without accompanying clinical context and demonstrate the importance of textual input alongside images.*

*It would also be helpful to reference work showing that, despite similar hallucination tendencies, LLMs perform strongly on standardized exams, for example:*

- *Gilson et al. How does ChatGPT perform on the USMLE? Implications for medical education and knowledge assessment. JMIR Med Educ. 2023 Feb 8;9:e45312.*
- *Novak et al. The pulse of artificial intelligence in cardiology: evaluating state-of-the-art LLMs for clinical cardiology. medRxiv. Preprint posted online on January 30, 2024.*

*These additions could situate the findings within a broader context of multimodal and high-stakes assessment.*

**Response:** We thank the reviewer for this valuable suggestion. We agree that the Discussion section benefits from a broader contextualization, particularly with respect to input formats and the evolving capabilities of multimodal models. At the current time, the diagnostic quality of multimodal models remains rudimentary, especially for images other than X-rays. As you suggested, we have added a paragraph to the Limitations section, where we stated that including imaging data in addition to the textual data would have most likely not led to a substantial improvement in model performance in our task–referring to the studies by Günay et al and Zeljkovic et al that you kindly mentioned.

In addition, we gladly added the references (Gilson et al, Novak et al) that you mentioned to the “Data Representation Affects LLM Performance” section of the Discussion to further strengthen our point that LLMs generally perform well when provided with concise and information-dense data but struggle with noisy and unprocessed clinical data.

*5. As an exploratory aside, it would be interesting to evaluate how the newest reasoning-focused models (eg, “o3” or “o4”) perform on this task. Although this is likely beyond the current scope, including a sentence to that effect in the manuscript’s Limitations section could guide future research.*

**Response:** We agree that in the fast-paced environment of LLM development, it is plausible that the newest reasoning-focused models might perform substantially better in our task than the reasoning models we used. We addressed this in the Limitations section.

*6. For consistency and precision, when describing model access in the “Large Language Models” section (and elsewhere in the text), the manuscript should explicitly cite the exact supplementary tables or materials (eg, “see Table S1 for model details and context sizes”) rather than referring generically to “the Supplementary.”*

**Response:** We agree that referring to specific supplementary tables and figures improves both clarity and precision. Accordingly, we have specified which supplementary tables and figures we are referring to throughout the manuscript.

*7. In the Statistical Methods subsection, rather than stating that nonnormally distributed data were compared using the Mann-Whitney U test “for nonnormally distributed continuous variables,” the phrasing could be tightened to “for variables departing from normality” or “for variables not following a normal distribution” to align with standard statistical terminology.*

**Response:** We thank the reviewer for this constructive suggestion. We have revised the phrasing in the “Statistical Analysis” subsection of the Methods to align with standard statistical terminology. Specifically, we now refer to the use of the Mann–Whitney *U* test for “variables departing from normality,” as recommended.

Changes made to the manuscript on our end:

- We made minor adjustments to the affiliations on the title page to align with newly introduced in-house guidelines.
- In Table 2 and Table S6, we replaced the previously reported age ranges (used in accordance with medRxiv’s data protection policy) with the actual patient ages, now presented as integer values.
- We replaced the term “non-LLM models” with “deterministic models” in the final paragraph before the Limitations section, as this terminology is more commonly used in recent literature and provides a more precise characterization.

## Abbreviations

LLM: large language model
